# Analysis of probe level patterns in Affymetrix microarray data

**DOI:** 10.1186/1471-2105-8-146

**Published:** 2007-05-04

**Authors:** Alexander C Cambon, Abdelnaby Khalyfa, Nigel GF Cooper, Caryn M Thompson

**Affiliations:** 1 Department of Bioinformatics and Biostatistics; School of Public Health and Information Sciences, University of Louisville, Louisville, Kentucky, USA; 2 Department of Pediatrics, Kosair Children's Hospital Research Institute, University of Louisville, Louisville, Kentucky, USA; 3 Department of Anatomical Science & Neurobiology, University of Louisville, Louisville, Kentucky, USA

## Abstract

**Background:**

Microarrays have been used extensively to analyze the expression profiles for thousands of genes in parallel. Most of the widely used methods for analyzing Affymetrix Genechip microarray data, including RMA, GCRMA and Model Based Expression Index (MBEI), summarize probe signal intensity data to generate a single measure of expression for each transcript on the array. In contrast, other methods are applied directly to probe intensities, negating the need for a summarization step.

**Results:**

In this study, we used the Affymetrix rat genome Genechip to explore variability in probe response patterns within transcripts. We considered a number of possible sources of variability in probe sets including probe location within the transcript, middle base pair of the probe sequence, probe overlap, sequence homology and affinity. Although affinity, middle base pair and probe location effects may be seen at the gross array level, these factors only account for a small proportion of the variation observed at the gene level. A BLAST search and the presence of probe by treatment interactions for selected differentially expressed genes showed high sequence homology for many probes to non-target genes.

**Conclusion:**

We suggest that examination and modeling of probe level intensities can be used to guide researchers in refining their conclusions regarding differentially expressed genes. We discuss implications for probe sequence selection for confirmatory analysis using real time PCR.

## Background

Microarray technology is a high-throughput method for studying the expression of thousands of genes simultaneously. Microarray data analysis is a multi-step procedure, and an overwhelming number of published methods exist for each step. Most popular methods for analyzing Affymetrix Genechip microarray data include background correction, normalization and summarization steps. For example, RMA [1] uses a model-based background correction, quantile normalization, and median polish summarization. These methods result in a robust and easily interpreted measure of expression for each probe set on an array. Subsequent tests for differential expression based on these methods have lower computing costs than probe level linear models.

A less widely used methodology incorporates probe information directly in the analysis for differential gene expression. For example, the affyPLM package [2] fits robust probe level linear models with fixed effects. An alternative is the probe level linear mixed model (hereafter referred to as PLLMM) introduced by Chu et al. [3]. This model does not include a summarization step, but uses log_2 _probe intensities directly in the model for tests of differential expression. In addition, the model includes a probe-by-treatment interaction term which, when significant, may indicate cross hybridization [3].

In this study, we explored probe response patterns in Affymetrix rat genome GeneChip microarrays. We also examined possible causes of variation among probes within transcripts such as probe affinities, homologies, and probe overlap. We discuss the value of using probe information directly in statistical models when the goal is to test for differential gene expression. To further investigate observed inconsistencies in probe response patterns between treatments, we conducted a BLASTN search using the complementary sequences from Affymetrix probe sets for selected genes.

## Results and discussion

**Figure 1:**
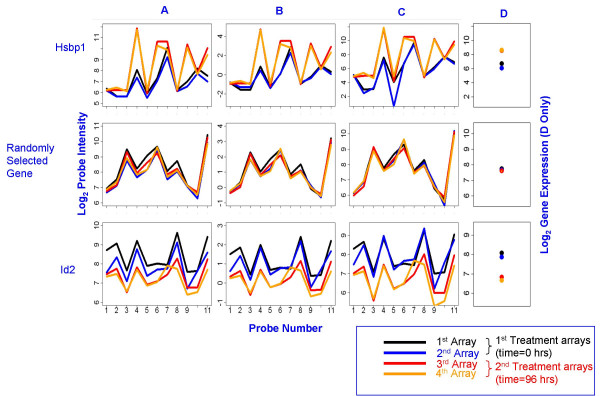
**Comparisons of probe level patterns across replicates and treatments for selected genes**. Each line color (in Panels A, B, and C) represents a probe level pattern on a specific array. Blue and black lines show probe level patterns on arrays from time = 0. Red and orange lines show probe level patterns on arrays for time = 96 hours. The first row shows probe level patterns for the highest up-regulated gene by fold change (Hspb1). The second row is a randomly selected gene. The third row is the most down-regulated gene by fold change. The x-axis for each plot in (Panels A, B, and C) is probe number (1 through 11). (Panel A) shows log_2 _of raw probe intensities by probe number. (Panel B) shows log_2 _array-centered intensities by probe number. The log_2 _quantile normalized mas-background-corrected probe intensities are shown in (Panel C). (Panel D) shows summary gene expression estimates using median polish. The summarization method used is exactly like the RMA method except that mas-background correction is used instead of rma background correction.

In Affymetrix arrays, all probes within a probe set should ideally estimate expression of the same gene. However, high levels of variation among probes, consistent across arrays, are often observed [4]. For example, the log_2 _probe intensities range from 6 to 12 in the probe set in the top row in Figure 1 Panel A. Other probe sets in Figure 1 have variation almost as extreme. Further examination shows that each probe set has a distinct profile which is mostly consistent across replicates. As a result, the variance for a single probe across replicates is an order of magnitude smaller than variance between probes within a replicate. Therefore, probe is usually included as a fixed effect in probe level linear mixed models. As demonstrated in Figure 1, the probe response pattern frequently varies considerably between treatments. This phenomenon is most obvious for Hsbp1 (GenBank: NM_031970) in Figure 1. Additional file 1, which shows probe patterns for the top 15 down-regulated genes, gives more examples. This phenomenon is modeled through inclusion of a probe by treatment interaction in the PLLMM [3].

**Figure 2:**
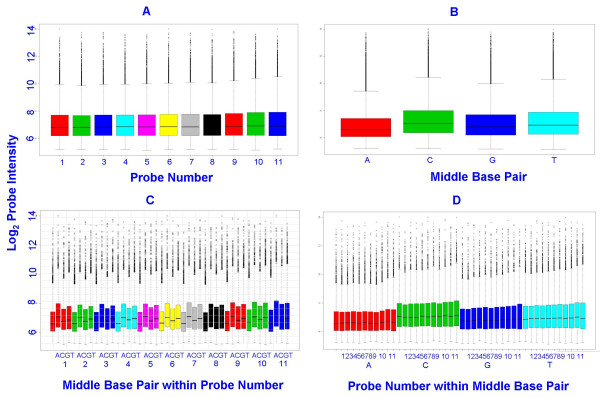
**Effect of middle base pair and probe number at gross array level**. The top left diagram (A) shows box plots by probe number (1–11) for all transcripts in array C. A slightly increasing trend can be seen. The top right diagram (B) shows box plots of probes in array C by middle base pair (A, C, G, and T) of the probe. The comparative levels are consistent with that described in the Naef and Magnasco [27] paper. The diagrams on the second row (C and D) show box plots of log_2 _probe intensities categorized by both probe number and middle base pair for all probes in array 3. The slight increasing trend at the array level is too weak to pick up in the plots of individual probe sets (Figure 1).

**Figure 3:**
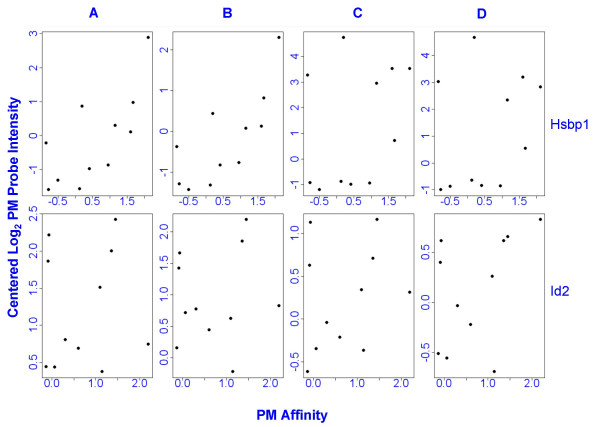
**Scatter plots of log_2 _array-mean-centered PM probe intensities vs. PM probe affinities**. The top row shows scatter plots for all four arrays (A,B,C,D) for Hsbp1, the number 1 up-regulated gene. The bottom row shows all four arrays for gene Id2, the top down-regulated gene. Affinities explain only a small part of variation between probes at the gene level. Affinities were calculated using the default method in Bioconductor package gcrma version 2.20. Affinities for perfect match probes are shown. Pearson correlation coefficients vary from 0.08 to 0.76 on the 8 scatter plots above. A scatter plot of affinities vs. log_2 _probe intensities (not shown) for all probes in array C are similar to the corresponding diagram in Wu et al. [5].

An effort to understand the sources of variability among probes within the probe set for each transcript is an area of much research. It has been suggested that some of the variation between probes for the same transcript can be explained by affinities, or position dependent base effects [5]. For example, Figures 2b, 2c, and 2d show the effect of the middle base pair in the probe sequence on log_2 _intensity at the gross array level. However, in this study, differences in probe affinities within transcripts, calculated using the gcrma package [6], accounted for only a small proportion of the total observed variation in probe intensities (Figure 3 and Additional file 2). Figures 2a and 2d also show a subtle increasing trend by probe number at the array level (probes are numbered in order of increasing distance from the 5' end). This is consistent with the fact that probe intensities are expected to be systematically lower at the 5' end of the probe set compared to the 3' end [7]. However, as with affinities, this phenomenon accounts for only a small proportion of total variation in probe intensities at the gene level (Figure 1).

Affinities are constant within a probe sequence, since they depend on the bases that make up a probe sequence. However, in some probe sets, the probe profile is consistent across replicates but not across treatments. For example the probe numbers 1, 5, 8, and 10 for gene heat shock 27 kDa protein 1, Hspb1 (GenBank: NM_031970), in the first row of Figure 1b display no treatment effect, while probe numbers 4, 6, 9, and 11 show extremely large treatment effects. A mixed model analysis along the lines of Chu et al. [3] confirms a statistically significant probe-by-treatment interaction for this gene.

Probe-by-treatment interactions can be due to cross hybridization [3]. The reason for this is as follows: since each 25 mer probe sequence in a probe set has a different sequence (except for overlap), partial homologies for each probe in a probe set will also be different. Therefore cross-hybridizing probes within a probe sequence will cross-hybridize to different sequences. Therefore if the signal due to cross-hybridization is large relative to the signal due to the target sequence of interest, the way in which these probes respond to treatment will be different according to the different sequences with which they are cross-hybridizing.

**Figure 4:**
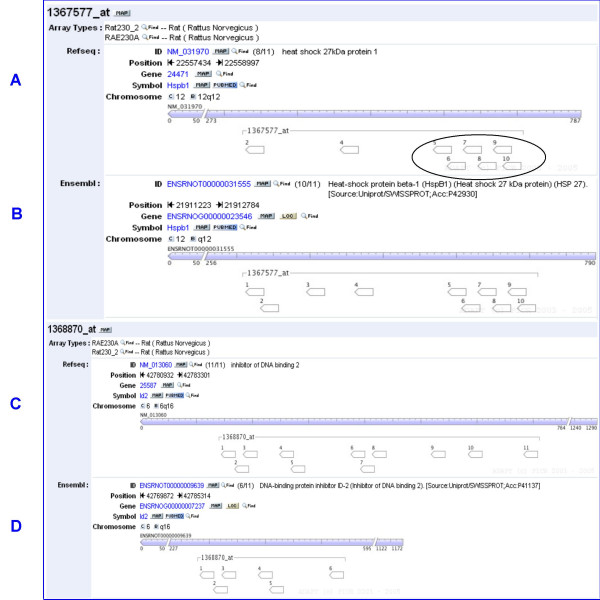
**The ADAPT tool**. The ADAPT tool shows positions of probes within the most highly differentially expressed up-regulated gene and the most highly differentially expressed down-regulated gene by fold change. The ADAPT tool used with RefSeq database confirms BLASTN results (Table 1) that probes 1,3, and 11 for gene Hsbp1 do not have perfect sequence matches with the gene. The diagram also elucidates the extent of probe overlap between probes 5 through 10 for Hsbp1 (A). The ADAPT used with the Ensemble database for Hsbp1 gives different results than the ADAPT tool used with the RefSeq database, however the results still show that probe 11 does not have a perfect sequence match with the gene (B). For gene Id2, the ADAPT tool shows that all probes have perfect sequence matches with the gene whether using RefSeq or Ensemble databases (C and D).

To investigate possible causes of cross hybridization, a BLAST nucleotide search was conducted using the complementary sequences from the Affymetrix probe sets for 8 selected genes; the search was restricted to the organism *Rattus Norvegicus*. Table 1 shows results for 2 of the selected genes. This search revealed that the sequences of 3 probes in the top up-regulated gene, Hsbp1 (GenBank: NM_031970), probe numbers 1, 3, and 11, do not match perfectly with any sequence in the gene. Further analysis using the ADAPT tool [8] and RefSeq [9] confirmed that the same 3 probes were missing (Figure 4). An ADAPT search of the same gene using Ensembl [10] shows a single non-matching probe (Figure 4). In comparison, similar BLAST and ADAPT searches for the top down-regulated gene, Id2 (GenBank: NM_013221), utilizing RefSeq or Ensembl, showed all 11 probe sequences matched the target gene perfectly (Figure 4). None of the other probe sets investigated had probe sequences which did not match the target gene perfectly. Dai et al. [11] address several issues in Affymetrix Genechip arrays, including probes which do not match perfectly the target sequence of interest.

**Table 1:** BLAST results for the highest up and down-regulated genes by fold change

			**Hsbp1**		**Highest Up-regulated Gene**						
			**Log 2 Array Mean centered**					**Perf. Seq.**	**Probe**	**Sequence**	

	**Probe**	**Probe**	**Probe Intensity by Array**					**w. target**	**Overlap**	**Homology**	

**Probe sequence**	**Name**	**Position**	**A**	**B**	**C**	**D**	**Affin.**	**gene**		**Perf.**	**Part.**

GGCAACTCAGCAGCGGTGTCTCAGA	1	309–333	-0.867	-0.771	-0.952	-0.860	0.953	0	5	1	33
TCAGAGATCCGACAGACGGCCGATC	2	329–353	-1.560	-1.322	-0.890	-0.646	0.123	1	0	1	2
GAGGAGCTCACAGTTAAGACCAAGG	3	392–416	-1.583	-1.287	-0.933	-0.991	-0.781	0	0	1	28
GATGAACATGGCTACATCTCTCGGT	4	458–482	0.862	0.429	4.728	4.665	0.198	1	0	3	28
AAGCAGTCACACAATCAGCGGAGAT	5	585–609	-1.310	-1.430	-1.198	-0.879	-0.500	1	6	3	10
GGAGATCACCATTCCGGTCACTTTC	6	604–628	0.105	0.119	3.522	3.191	1.606	1	3	3	6
TTCGAGGCCCGTGCCCAAATTGGAG	7	626–650	2.880	2.293	3.521	2.828	2.127	1	5	3	5
TGGAGGCCCAGAGTCGGAACAGTCT	8	646–670	-0.974	-0.827	-0.993	-0.841	0.412	1	4	3	16
GTCTGGAGCCAAGTAGAAGCCTTCA	9	667–691	-0.215	-0.384	3.274	3.027	-0.843	1	12	3	33
TAGAAGCCTTCAGCTTGCTACCCAT	10	680–704	0.970	0.812	0.711	0.546	1.685	1	0	3	21
TCCCTCTCTGTCAATCTGATATGCT	11	727–745	0.303	0.069	2.940	2.345	1.162	0	NA	0	19

			**Id2**		**Highest Down-regulated Gene**						

TGGACGACCCGATGAGTCTGCTCTA	1	144–168	1.507	0.623	0.342	0.258	1.1012	1	1	4	3
ACAACATGAACGACTGCTACTCCAA	2	168–192	1.861	1.423	0.630	0.399	-0.082	1	10	4	9
GCTACTCCAAGCTCAAGGAACTGGT	3	183–207	0.437	0.156	-0.616	-0.511	-0.124	1	0	4	45
ATCCTGCAGCACGTCATCGATTATA	4	248–272	2.003	1.848	0.710	0.615	1.358	1	5	4	17
TTATATCTTGGACCTGCAGATCGCC	5	268–292	0.688	0.442	-0.215	-0.221	0.6038	1	0	4	37
TGAACACGGACATCAGCATCCTGTC	6	375–399	0.806	0.775	-0.041	-0.034	0.3126	1	8	4	32
ATCCTGTCCTTGCAGGCGTCTGAAT	7	392–416	0.741	0.822	0.310	0.826	2.1972	1	4	3	15
GAATTCCCTTCTGAGCTTATGTCGA	8	413–437	2.422	2.189	1.162	0.656	1.4553	1	0	3	41
TTCTCTTTTTCTTTTGCACAACAAG	9	518–542	0.375	-0.217	-0.369	-0.691	1.1409	1	0	3	97
TGTTATCAACCATTTCACCAGGAGA	10	587–608	0.434	0.713	-0.350	-0.557	0.0674	1	0	3	40
GGCCTGGACTGTGATAACCGTTATT	11	683–707	2.214	1.663	1.130	0.615	-0.061	1	NA	3	19

The BLAST search was restricted to organism *Rattus norvegicus*, however there is some duplication due to presence of multiple entries in some databases used in BLAST. Note that not all probes for Hsbp1 match perfectly with the target gene.

If a probe sequence does not match the target sequence of interest, it will increase the signal due to cross hybridization relative to the signal due to hybridization to the target sequence of interest. The non-matching probe sequences for Hsbp1 (GenBank: NM_031970) may partly explain the unusually high probe-by-treatment interaction for this gene.

**Figure 5:**
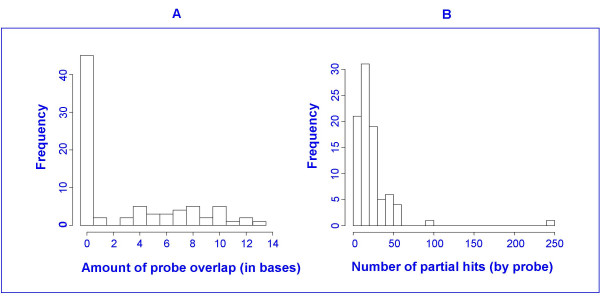
**Histograms of BLAST results conducted on 8 selected genes**. BLAST results were conducted on four up-regulated genes and four down-regulated genes. The histogram on the left (A) shows the amount of probe overlap for all probes in the 8 probe sets. Slightly more than half of the probes had no overlap. The rest of the probes had overlap of between 1 and 13 bases. The histogram on the right (B) shows the number of partial homologies for each of the probes. The median number of partial homologies over all 88 probes submitted to BLAST was 17, and first and third quartiles were 11 and 28, respectively. The search was restricted to organism *Rattus norvegicus*.

Sequence similarity results from the BLAST search showed that almost all probe sequences had sequence similarities with alignment-length greater than 14 base pairs (bp). In fact the "E" score in BLAST for alignment lengths of 14 bp was 5.4, meaning that, on average, 5.4 alignment lengths of 14 bp are to be expected by chance alone based on the search criteria and the number of sequences in the search database [12]. The E score for alignment lengths of 13 bp is 22. These alignment-lengths are well within the range that can contribute to cross-hybridization [13]. Thus, it is to be expected that with 25 mer probes for rat or human genomes, cross hybridization is a contributing factor and needs to be accounted for in any proposed statistical model. In fact, the probe design criteria used by Stekel [14] for 30 mer probes reject probes having sequence similarities with alignment lengths of 15 bp or greater. Figure 5 shows histograms of partial sequence homologies and probe overlap for the 88 probes from the 8 selected genes from the BLAST search. The median number of partial sequence homologies for a probe in this group was 17, and the first and third quartiles were 11 and 28 respectively. The results probably overestimate the actual number of partial hits due to redundancies in BLAST databases.

Attempts by the authors to directly model log_2 _probe intensity as a function of cross hybridization using information solely based on the BLAST searches proved inconclusive. Adjusted log_2 _PM probe intensity was modeled as a function of number of partial BLAST hits for a probe and PM probe affinities. The PM affinity term was significant as expected. The number of partial BLAST hits was not significant. We also modeled absolute deviation from average treatment effect as a function of number of partial BLAST hits. Regulation (up vs. down-regulated) was also added as a factor for both models. The reason the results were inconclusive may be due to the complexity of the mechanisms involved in the cross hybridization process and the numbers of sequences from different non-target genes that can cross hybridize with a single probe. However, a free energy position dependent nearest neighbor (PDNN) model based on PM sequences has been used to model log_2 _probe intensity as a function of gene specific hybridization, non specific hybridization, and background [15]. This model has the advantage that it does not rely on mismatch (MM) probes to model cross hybridization. Zhang et al. [15] demonstrate that it is inappropriate to use the mismatch probe to model cross-hybridization of the corresponding perfect match probe, since the mechanism for cross-hybridization is different. The results show that the "model is able to explain most of the variations of probe signals in a probe set" [13]. However, as indicated by Wu, Carta, Zhang [13], even this model does not take into account all factors that contribute to probe intensity, such as RNA secondary structure.

The PLLMM models probe variation and treatment variation separately. Therefore an advantage of the PLLMM over summarization methods (which include PDNN) is that it includes a probe-by-treatment interaction term which can indicate presence of cross hybridization [3]. Also, since it includes probe as a fixed effect, it adjusts for differences among probes, regardless of their cause. The strong effect of cross hybridization on probe intensity has been demonstrated by Zhang et al. [15]. In this data set, using the PLLMM to analyze the list of top 30 up and down regulated genes supports this observation. Nine of the 30 genes had p-values less than .0001 for interaction, and 18 out of 30 had p values below .01 for interaction. Furthermore, this finding is consistent with the BLAST search results discussed above. Summary methods, such as RMA, provide no mechanism for detecting this probe-treatment interaction. It is interesting that the gene with the highest up-regulated fold change, heat shock 27 kDa protein 1, Hspb1, (GenBank: NM_031970), as determined by RMA with MAS background correction, also has by far the largest probe-treatment interaction, and that, as stated earlier, four of the probes in the probe set show no treatment effect.

For differentially expressed genes identified in microarray experiments, validation using other techniques, such as RT-PCR is required. However, there are often discrepancies between results obtained from microarray experiments and RT-PCR analysis. For example, Rejeevan et al. [16] were unable to achieve consistent validation using RT-PCR for genes showing less than a four-fold difference in a microarray experiment. Recent studies have shown that agreement between microarray and RT-PCR results depends to some extent on the background, normalization and summarization methods used to calculate gene expression [17,18]. The potential for probe by treatment interactions in microarray experiments may also partially explain difficulty in validating results. The presence of variation among probes in probe sets, and inconsistency in probe response patterns between treatments suggests that care must be taken in designing primers for RT-PCR. Carter et al. [19] provide guidance for improving consistency of probe sequences across platforms.

## Conclusion

Genome-wide expression profiling with microarrays generates a tremendous amount of data. It is critical to develop acceptable tools and guidelines for data analysis. The signal intensity of probe sets for each gene should be related to the abundance of the transcript, which can be used to quantify the level of gene expression. However, this signal can be distorted due to many factors, including cross-hybridization. Although, the inclusion of mismatch probes was an attempt to adjust for non-specific binding, it has now been established that the mechanism of cross hybridization is different for mismatch probes than for perfect match probes. We have demonstrated that tests for differential expression based on the most widely used summarization methods alone may be misleading, since they provide no means for examining this effect. We suggest that examination of probe level patterns and PLMM analysis can be used to identify genes potentially affected by these issues. These genes should be investigated further in order to make appropriate conclusions regarding differential expression.

## Methods

### Culture of RGC-5 cells

Cultures of RGC-5 cells were maintained in growth medium containing low-glucose Dulbecco's modified Eagle's medium (DMEM) containing 10% fetal calf serum (FCS), 100 U/mL penicillin, and 100 μg/mL streptomycin (Sigma-Aldrich, St. Louis, MO) in a humidified atmosphere of 95% air and 5% CO_2 _at 37°C, as described by Krishnamoorthy et al. [20]. To induce apoptosis, the growth medium was withdrawn (serum starvation) and cells were maintained in DMEM for 0, 4 days. Total RNA was extracted from each biological replicate for each time point and maintained at -80°C until used for analysis.

Total RNA was extracted from the RGC-5 cells using spin columns (RNeasy; Qiagen, Valencia, CA), followed by DNase treatment according to the manufacturer's instructions. The quantity and purity of total RNA for samples were analyzed with spectrophotometry readings at 260/280 nm. The integrity of intact total RNA was verified with an Agilent 2100 Bioanalyzer (Agilent Technologies, Palo Alto, CA). RNA samples were each prepared to a concentration of 25 ng/μl in parallel to a 6000 RNA ladder (Ambion, Houston, TX). The range of 28S/18S ribosomal RNA was typically 1.8 to 2.1.

Purified total RNA (20 μg) was converted to first strand cDNA using a T7-linked oligodeoxythymidylic acid primer (Genset, La Jolla, CA) followed by second strand synthesis (Invitrogen Corporation, Carlsbad, CA). The cDNA was then converted to labeled cRNA using T7 RNA polymerase in the presence of biotinylated UTP and CTP (Enzo Diagnostics, Farmingdale, NY). The labeled cRNA was purified on a RNeasy column (Qiagen Valencia, CA), fragmented, and used to make up the hybridization cocktail containing control oligonucleotide B2 and four control bacterial and phage cRNAs (BioB, BioC, BioD, cre). Labeled cRNA (15 μg) were hybridized to Affymetrix Rat GeneChips (Affymetrix, Santa Clara, CA) using standard conditions in an Affymetrix fluidics station.

Samples from the biological replicates for each group were hybridized to a set of two independent Affymetrix GeneChip Rat arrays using the protocol described in the Affymetrix Expression Analysis. Hybridization was performed at 42°C for 16 h; followed by washing, staining, signal amplification with biotinylated antistrepavidin antibody, and the final staining step.

### Affymetrix gene array

Affymetrix rat genome GeneChips (Array 230A) were used to compare signal intensity profiles of apoptotic and non-apoptotic retinal ganglion cells (RGC-5). The cells were induced into apoptosis by serum deprivation for 96 hours. Two biological replicates were used for each time point.

In Affymetrix GeneChips arrays, a transcript is represented by a set of 11–20 probe pairs, each consisting of a perfect match (PM) and a mismatch (MM) probe of 25 base pairs. Probe sets in the Array 230A consist of 11 probe pairs. The PM probes that represent a gene are designed to hybridize to different regions of the RNA for the corresponding gene. These probes act as multiple detectors of the gene. The MM probe within each pair is created by changing the middle base of the corresponding PM probe to its complementary base. The original intent of including the MM probes was to account for nonspecific hybridization.

### Microarray quality analysis

The Bioconductor packages "affy" [21] and "affyPLM" were used to generate images, histograms, box plots, degradation plots, and scatter plots to evaluate the quality of the hybridized arrays.

### Data analysis

To calculate probe set expression values for each probe set, MAS background, quantile normalization, and median polish [1] for the PM probes only were used on each array. A list of differentially expressed genes were identified using an empirical Bayes method and a false discovery rate (FDR) correction [22] with cut-off of p = 0.05. Of the 14,000 genes on the Array 230A, 23 differentially up-regulated genes and 47 down-regulated genes with RefSeq accession numbers were identified. The up and down regulated genes were ranked separately by fold change, and the 1^st^, 5^th^, 10^th^, and 15^th ^ranked genes from each set were profiled to enable visual examination of probe level behavior across replicates and treatments. For comparison, probe sets from 2 randomly selected genes across the rat genome were also profiled.

### BLAST probe set sequencing

The genomic DNA sequences from each perfect match probe in a sample of probe sets were submitted to the National Center for Biotechnology Information (NCBI) database [23]. Using the BLAST tool, the number of perfect and partial sequence matches was recorded for each probe set. The position of each probe in the gene sequence was recorded using the BLAST tool and Bioconductor [24]. This enabled the amount of overlap in sequence between adjacent probes to be calculated.

### Probe set affinity calculations

Affinities for each probe in the selected sets were calculated using the method of Irizarry and Wu [5]. For each probe set affinity-corrected and log_2 _array-mean centered probe intensities were compared. Bioconductor [24] packages affy [25], gcrma [13], and limma [26], as well as the SAS PROC MIXED procedure (Version 9.1, SAS Institute), were used for all gene and probe computations.

## Authors' contributions

ACC performed data analysis and contributed to the manuscript preparation. AK carried out the microarray experimental work, was involved in microarray analysis, and manuscript preparation. CMT was involved in data analysis and manuscript preparation. The microarray data was obtained from the KBRIN microarray core laboratory supported by NIH:P20RR16481 (NGFC).

## Supplementary Material

Additional file 1 **Comparisons of probe level patterns across replicates and treatments for the top 15 down-regulated genes**. The line colors follow the same key as Figure 1. Each line color represents a probe level pattern on a specific array. Blue and black lines show probe level patterns on arrays from time = 0. Red and orange lines show probe level patterns on arrays for time = 96 hours. As in Figure 1 Panel B, the plots all show log_2 _array-centered intensities by probe number. Click here for file

Additional file 2 **Additional scatter plots of log_2 _array-mean-centered PM probe intensities vs. PM probe affinities**. The top row shows scatter plots for all four arrays (A,B,C,D) for the 1^st ^and 2^nd ^up-regulated genes. The second row shows scatter plots for the 3^rd ^and 4^th ^up-regulated genes. The third row shows scatter plots for the 5^th ^and 6^th ^up-regulated genes. The last row shows the scatter plots for the 7^th ^and 8^th ^genes. Click here for file

## References

[B1] Irizarry RA, Hobbs B, Collin F, Beazer-Barclay YD, Antonellis KJ, Scherf U, Speed TP (2003). Exploration, normalization, and summaries of high density oligonucleotide array probe level data. Biostatistics.

[B2] Bolstad BM affyPLM: Methods for fitting probe-level models..

[B3] Chu TM, Weir B, Wolfinger R (2002). A systematic statistical linear modeling approach to oligonucleotide array experiments. Math Biosci.

[B4] Li C, Wong WH (2001). Model-based analysis of oligonucleotide arrays: expression index computation and outlier detection. Proc Natl Acad Sci U S A.

[B5] Wu Z, Irizarry RA, Gentleman R, Martinez-Murillo F, Spencer F (2004). A model based background adjustment for oligonucleotide expression arrays. Journal of the American Statistical Association.

[B6] Wu C, Irizarry R, with contributions from Macdonald J, Gentry J (2005). gcrma:Background Adjustment Using Sequence Information..

[B7] Gautier L, Irizarry R, Cope L, Bolstad BM Description of affy (affy vignette). http://www.bioconductor.org/repository/devel/vignette/affy.pdf.

[B8] Leong HS, Yates T, Wilson C, J. MC (2005). ADAPT: A database of Affymetrix Probesets and Transcripts. Bioinformatics.

[B9] Pruitt KD, Tatusova T, Maglott DR (2005). NCBI Reference Sequence (RefSeq): a curated non-redundant sequence database of genomes, transcripts and proteins. Nucleic Acids Res.

[B10] Birney E, Andrews D, Caccamo M, Chen Y, Clarke L, Coates G, Cox T, Cunningham F, Curwen V, Cutts T, Down T, Durbin R, Fernandez-Suarez XM, Flicek P, Graf  S, Hammond M, Herrero J, Howe K, Iyer V, Jekosch K, Kahari A, Kasprzyk A, Keefe D, Kokocinski F, Kulesha E, London D, Longden I, Melsopp C, Meidl P, Overduin B (2006). Ensembl 2006. Nucleic Acids Res.

[B11] Dai M, Wang P, Boyd AD, Kostov G, Athey B, Jones EG, Bunney WE, Myers RM, Speed TP, Akil H, Watson SJ, Meng F (2005). Evolving gene/transcript definitions significantly alter the interpretation of GeneChip data. Nucleic Acids Res.

[B12] Karlin S, Altschul SF (1990). Methods for assessing the statistical significance of molecular sequence features by using general scoring schemes. Proc Natl Acad Sci U S A.

[B13] Wu C, Carta R, Zhang L (2005). Sequence dependence of cross-hybridization on short oligo microarrays. Nucleic Acids Res.

[B14] Stekel D (2003). Microarray bioinformatics.

[B15] Zhang L, Miles MF, Aldape KD (2003). A model of molecular interactions on short oligonucleotide microarrays. Nat Biotechnol.

[B16] Rajeevan MS, Vernon SD, Taysavang N, Unger ER (2001). Validation of array-based gene expression profiles by real-time (kinetic) RT-PCR. J Mol Diagn.

[B17] Qin LX, Beyer RP, Hudson FN, Linford NJ, Morris DE, Kerr KF (2006). Evaluation of methods for oligonucleotide array data via quantitative real-time PCR. BMC Bioinformatics.

[B18] Millenaar FF, Okyere J, May ST, van Zanten M, Voesenek LA, Peeters AJ (2006). How to decide? Different methods of calculating gene expression from short oligonucleotide array data will give different results. BMC Bioinformatics.

[B19] Carter SL, Eklund AC, Mecham BH, Kohane IS, Szallasi Z (2005). Redefinition of Affymetrix probe sets by sequence overlap with cDNA microarray probes reduces cross-platform inconsistencies in cancer-associated gene expression measurements. BMC Bioinformatics.

[B20] Krishnamoorthy RR, Agarwal P, Prasanna G, Vopat K, Lambert W, Sheedlo HJ, Pang IH, Shade D, Wordinger RJ, Yorio T, Clark AF, Agarwal N (2001). Characterization of a transformed rat retinal ganglion cell line. Brain research.

[B21] Affymetrix (2004). Affymetrix Microarray User Guide.

[B22] Benjamini Y, Hochberg Y (1995). Controlling the false discovery rate: a practical and powerful approach to multiple testing. Journal of the Royal Statistical Society Series B Methodological.

[B23] Altschul S.F. GW National Center for Biotechnology Information; BLAST.

[B24] Bioconductor. http://www.bioconductor.org.

[B25] Irizarry R, Gautier L, Bolstad BM, Miller C, with contributions from Astrand M, Cope LM, Gentleman R, Gentry J, Halling C, Huber W, MacDonald J, Rubinstein BIP, Workman C, Zhang J (2005). affy: Methods for Affymetrix Oligonucleotide Arrays..

[B26] Smyth GK (2005). Limma: Linear Models for Microarray Data.

[B27] Naef F, Magnasco MO (2003). Solving the riddle of the bright mismatches: labeling and effective binding in oligonucleotide arrays. Phys Rev E Stat Nonlin Soft Matter Phys.

