# Supplementary figures and images for: Analysis of probe level patterns in Affymetrix microarray data
Source: BMC Bioinformatics. 2007 May 4;8:146. doi: 10.1186/1471-2105-8-146 (PMC1884176; doi:10.1186/1471-2105-8-146)

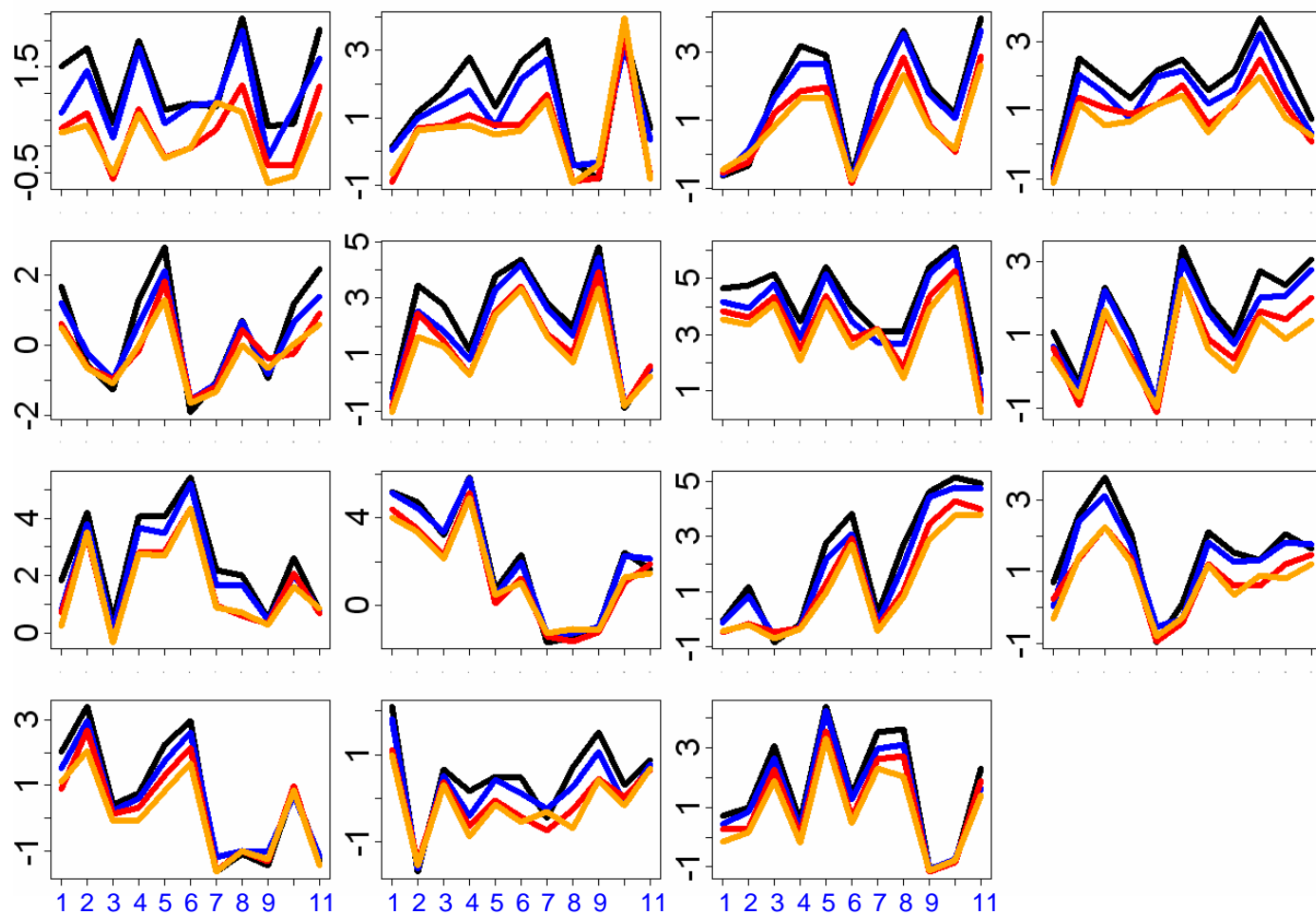

Probe Number

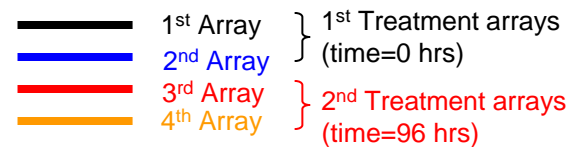

Supplement: Additional file 1 — Comparisons of probe level patterns across replicates and treatments for the top 15 down-regulated genes. The line colors follow the same key as Figure 1. Each line color represents a probe level pattern on a specific array. Blue and black lines show probe level patterns on arrays from time = 0. Red and orange lines show probe level patterns on arrays for time = 96 hours. As in Figure 1 Panel B, the plots all show log2 array-centered intensities by probe number. [file 1471-2105-8-146-S1.pdf]

Centered Log<sub>2</sub> PM Probe Intensity

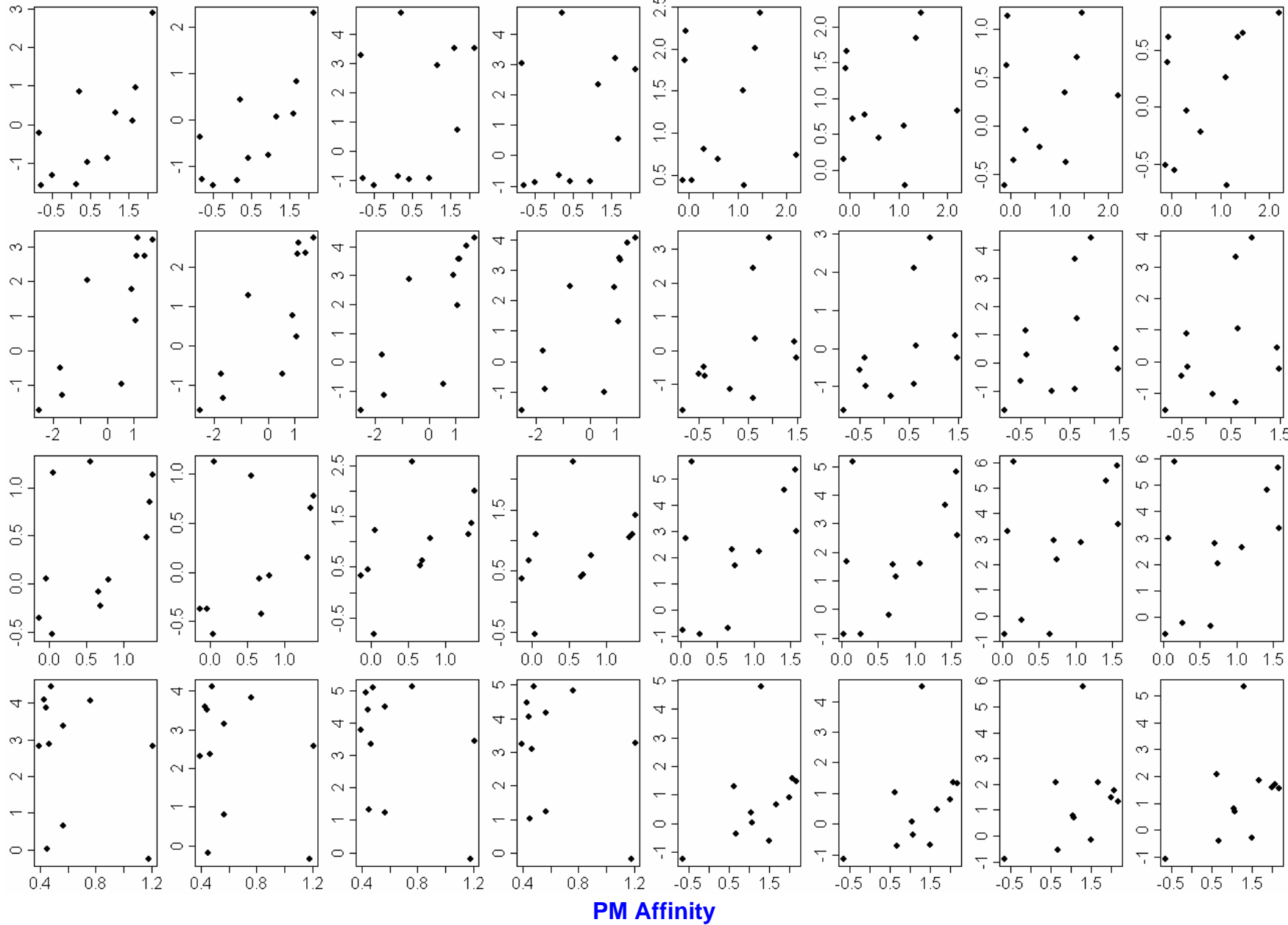

Supplement: Additional file 2 — Additional scatter plots of log2 array-mean-centered PM probe intensities vs. PM probe affinities. The top row shows scatter plots for all four arrays (A,B,C,D) for the 1st and 2nd up-regulated genes. The second row shows scatter plots for the 3rd and 4th up-regulated genes. The third row shows scatter plots for the 5th and 6th up-regulated genes. The last row shows the scatter plots for the 7th and 8th genes. [file 1471-2105-8-146-S2.pdf]
